# Physiological mechanism of melatonin attenuating to osmotic stress tolerance in soybean seedlings

**DOI:** 10.3389/fpls.2023.1193666

**Published:** 2023-05-27

**Authors:** Mohammad Shah Jahan, Chang Jiang Zhao, Li Bo Shi, Xiu Ren Liang, Dilfuza Jabborova, Jamal Nasar, Xun Bo Zhou

**Affiliations:** ^1^ Guangxi Key Laboratory of Agro-environment and Agro-products Safety, Key Laboratory of Crop Cultivation and Physiology, College of Agriculture, Guangxi University, Guangxi, Nanning, China; ^2^ Department of Horticulture, Faculty of Agriculture, Sher-e-Bangla Agricultural University, Dhaka, Bangladesh; ^3^ MAP Division (Shandong) of Sinochem Agriculture Holdings, Jinan, China; ^4^ Guangxi Ecoengineering Vocational and Technical College, Liuzhou, China; ^5^ Laboratory of Medicinal Plants Genetics and Biotechnology, Institute of Genetics and Plant Experimental Biology, Academy of Sciences of Uzbekistan, Tashkent, Uzbekistan

**Keywords:** Aquaporins, ABA metabolism, soybean, drought, climate change, water use efficiency

## Abstract

Drought is one of the most significant abiotic stress threatening to crop production worldwide. Soybean is a major legume crop with immense economic significance, but its production is highly dependent on optimum rainfall or abundant irrigation. As the global climate changes, it is more important to find solutions to make plants more resilient to drought. The prime aimed of the study is to investigate the effect of melatonin on drought tolerance in soybean and its potential mechanisms. Soybean seedlings were treated with 20% polyethylene glycol 6000 (PEG 6000) and subjected to osmotic stress (14 days) with or without 100 μM melatonin treatment. Our results revealed that melatonin supplementation significantly mitigated PEG-induced growth retardation and increased water absorption ability. Foliar application of melatonin also increased gas exchange and the chlorophyll fluorescence attributes by the mitigation of the osmotic-induced reduction of the reaction activity of photosystems I and II, net photosynthetic rate (Pn), stomatal conductance (Gs), transpiration rate (Tr), electron transport activity, and photosynthetic efficiency. In addition, PEG-induced elevated production of reactive oxygen species (ROS) and malondialdehyde (MDA) content were significantly reversed by melatonin treatment. Equally important, melatonin boosted the antioxidant activities of soybean plants. Moreover, osmotic stress substantially increased abscisic acid (ABA) accumulation in roots and leaves, while melatonin-received plant leaves accumulated less ABA but roots content higher ABA. Similarly, melatonin significantly suppressed ABA biosynthesis and signaling gene expression in soybean exposed to drought stress. Furthermore, osmotic stress significantly suppressed plasmalemma (*GmPIPs*) and tonoplast aquaporin (*GmTIPs*) genes expression, and their transcript abundance was up-regulated by melatonin co-addition. Taken together, our results indicated that melatonin potentially improves drought tolerance of soybean through the regulation of ABA and aquaporin gene expression, increasing photosynthetic efficiency as well as enhancing water uptake efficiency.

## 1 Introduction

Soybean (*Glycine max* L.) is an important oil seed legume and forage crop, rich in protein (40%) and edible oil (20%), cultivated globally for its valuable seed composition (Sharmin et al., 2020). The annual global soybean production in 2019 was estimated to exceed 333 million tonnes (FAOSTAT, 2019). Soybean is mainly grown in tropical, subtropical, and temperate regions (FAO, 2021). It is very sensitive to water scarcity and suffers greatly in terms of growth and development under water deficit conditions (Bortoluzzi et al., 2020). Consequently, global warming and changes in precipitation patterns pose a significant threat to soybean production, especially in areas lacking of rainwater or irrigation (Cotrim et al., 2021). It is well known that soybean yield can be reduced under dry conditions (>50%), resulting in substantial economic losses for farmers and growers (Soltani et al., 2017). Hence, drought is a significant climatic risk requiring effective mitigation strategies to sustain the global soybean supply. Over the past two decades, surface water and groundwater have been rapidly declining in China, approaching the threshold of internationally recognized water resources (Chai et al., 2014). Osmotic stress has profound negative effect on plants growth and development as well as it substantially decrease the pigment contents (Altaf et al., 2022). Malondialdehyde (MDA) is a product of membrane peroxidation and is commonly used as a prototypical symbol of membrane damage in stressed cells (Wang et al., 2018). The key antioxidant enzymes superoxide dismutase (SOD), peroxidase (POD), and glutathione-s-transferase (GST) are actively participated against stress environments (Alharby and Fahad, 2020; Alam et al., 2022). The increased antioxidant enzyme activity can decrease MDA accumulation and reduce stress-induced cellular structural damage (Ahmad et al., 2014). Osmotic regulation is an essential physiological process for plants in response to water deprivation (Iqbal et al., 2018). The most important osmolytes in plants are soluble saccharides and proline, which are required to maintain stable cellular outcomes under osmotic stress conditions (Dutta et al., 2019). Under abiotic stress conditions, increased production of reactive oxygen species (ROS) changes the phytohormone proteome and regulates the gene expression (Dalal et al., 2018). Drought stress can cause an imbalance in intracellular ROS production, leading to modulates oxidative stress responses (Raja et al., 2017).

Multiple endogenous hormones and metabolic components collaborate to govern the growth and development of plants (Dong et al., 2019). Abscisic acid is an important phytohormone that plays a key role in stress responses in different plants, generally elevates in plants exposed to drought stress which controls stomatal opening and closing and enhances the hydrophobic water holding capacity (Hossain et al., 2015). Hence, decrease in stomatal opening causes lowered CO_2_ assimilation and reduced photosynthesis activity, which imparts the growth and development of plants (Mutava et al., 2015; Cohen et al., 2021). Aquaporins (AQPs) are tiny integral membrane proteins that enable water transport across cells and contribute to water homeostasis in plants. Numerous physiological and genetic studies have demonstrated that AQPs are responsible for short-term alterations in root hydraulics and leaf water relations (Shivaraj et al., 2021). Among the recognized AQP subfamilies, members of the plasma membrane intrinsic protein (*PIP*) and the tonoplast intrinsic protein (*TIP*) are widely reported for their significant role under stress conditions (Shivaraj et al., 2021; Sudhakaran et al., 2021). Arabidopsis plants exposed to water deficit condition have shown down regulation of *TIPs* expression in leaves but only one *PIP (PIP2;5)* showed higher expression (Alexandersson et al., 2010). The expression of the strawberry *FvPIP2;1* and *FvPIP2;2* genes was observed both in leaves and roots, where, *FvPIP1;1* expression was only found in roots under drought stress conditions (Šurbanovski et al., 2013). Unlike *PIPs*, barley leaves exposed to 14 days of drought stress has shown differential expression of *TIPs (* Kurowska et al., 2019). Interestingly, PIPs and TIPs are widely known aquaporins involved in transporting H_2_O_2_ in a stressful environment (Carvalho, 2008). The efficacy of *AtTIP1;1*, *AtTIP1;2*, and *AtTIP2;3* to transport H_2_O_2_ has been investigated by applying a yeast experiment (Bienert et al., 2007). In another study, *CsTIP2; 1* overexpressed tobacco plants increased drought tolerance, and it’s strongly associated with the restriction of H_2_O_2_ production. These investigations show that *TIPs*, are involved in the implication of H_2_O_2_ into the vacuoles or strengthen the antioxidative system to quench the excess ROS, ultimately offering drought resilience to plants.

Many techniques, such as candidate genes, breeding, and multi-omics approaches, could be used to develop transgenic soybean lines which are more tolerant to drought stress (Dubey et al., 2018). Despite the strategies mentioned above, the application of exogenous hormones and/or biostimulants offers a simpler and more cost-effective strategy to improve the resilience of plants to adverse consequences of different abiotic stresses, including drought stress (Khan et al., 2019; Shahzad et al., 2023). Among them, melatonin is a ubiquitous master-class plant growth regulator, playing significant role in the regulation of plant growth and development under stressful environments (Tiwari et al., 2021). Melatonin promotes root and shoot formation, inhibits chlorophyll breakdown/degradation, slows the senescence process, and reduces ROS generation (Arnao and Hernández-Ruiz, 2019). Melatonin markedly improved the ability of tomato seedlings to withstand drought conditions by improving stomatal conductivity, photosynthetic rate, quantum PSII (Fv/Fm), transpiration rate, and electron transport (Liu et al., 2015). Besides this, melatonin increases the expression of abscisic acid (ABA) catabolism genes (two *CYP707* monooxygenases) while decreasing the expression of *NCED*, a vital enzyme in ABA production, leading to sharp reduction in ABA concentrations under stressful circumstances (Zhang et al., 2014).

The implication of melatonin on aquaporin gene expression has also been studied in plants, but its findings are very limited. Though aquaporins are one of the core transporters controlling the hydraulic conductance of roots (Deshmukh et al., 2017), and melatonin assists in mitigating osmotic stress, so melatonin and aquaporins are inextricably linked with each other (Rajora et al., 2022). Supplementation with melatonin improved the efficiency of root water absorption in rice through the regulation of plasma membrane intrinsic proteins (PIPs). Another study on maize showed that melatonin application enhances entire hydraulic conductance in particular roots through upregulation of *PIPs* expression (Qiao et al., 2020; Tiwari et al., 2021). However, in-depth studies on the dynamics of aquaporin expression under osmotic stress in the presence of melatonin are inadequate. Abscisic acid generally plays an essential function in AQP modulation and root water uptake in plants confronting diverse nitrogen forms and/or water deficit conditions (Parent et al., 2009). Melatonin’s impact on ABA dynamics, including how ABA content controls AQP expression and water uptake in plants during drought stress, is still not fully understood. The current study sought to investigate the potential functions of melatonin in enhancing drought tolerance, and the mechanism of melatonin-induced enhanced resilience to drought. We assumed that melatonin could facilitate higher accumulation of ABA in roots than leaves in soybean and trigger the expression of AQPs under drought stress conditions and also justified the dynamics functions of melatonin in regulating ABA and AQPs under drought stress condition.

## 2 Materials and methods

### 2.1 Plant materials and growth conditions

Soybean seeds were procured from Guangxi University, followed by sterilization in 20% ethanol, and placed in an incubator for germination. The germinated seeds were sown in plastic trays (32 holes) filled with growth substrates and cultured at the Guangxi University greenhouse. The flow rate, humidity, and temperature in the chamber were kept constant at 250 mol m^-2^ s^-1^, 65%, and 26°C, respectively. At the second true leaf stage seedlings were transplanted in new pots (the diameter of top and bottom were in 24 cm and 23 cm, respectively; 9 cm in height; water outlet at the bottom) containing 3.5 kg mixture of humus soil and sand (1:1). Soybean seedlings were watered with distilled water (50 mL/pot) as a control or with polyethylene glycol 6,000 (PEG-6000, Solarbio co., Beijing, China) at concentrations of 0%, 10%, 20%, and 30% concentrations (50 mL/pot). We applied PEG-6000 to induce osmotic stress. Each group consisted of three replications. The plants underwent numerous physiological and biochemical evaluations following the end of each treatment cycle (14 DAT, data is not shown). Finally, 20% polyethylene glycol (PEG) was finalized as the optimal concentration for further investigations.

In addition, the concentration of melatonin was selected before the final experiment. Plants in the control group were well watered with a ½ strength Hoagland nutrient solution, in the melatonin group plants were watered with a ½ strength Hoagland nutrient solution and foliar sprayed with 100 mL (100 µM concentration) melatonin solution and melatonin was applied every three alternate day (4 times and continue until 12 days of treatment). PEG treatment group were watered with a ½ strength Hoagland nutrient solution plus 20% PEG 6,000, and PEG + melatonin group were watered with a ½ strength Hoagland nutrient solution plus 20% PEG 6,000 plus 100 mL (100 µM concentration) melatonin solution. The designed parameters were recorded on the 14^th^ day after the treatment, and samples were harvested for biochemical and gene expression analysis, followed by storage at -80 °C. At least three replicates were performed for each treatment and 32 plants were grown randomly for each treatment.

### 2.2 Determination of biomass and relative water content

At least five seedlings from each treatment group were washed with running water. Excess water was removed from the plant’s surface by tissues before the stems and roots were separated. Following that, the fresh weight (FW) was calculated. The biomass and root-to-shoot ratio of soybean were determined. To determine the relative water content (RWC) of tissues (Jahan et al., 2019), leaves or roots with a specified FW were immersed in distilled water for 5 hours to saturation. The saturated weight (SW) was determined after the absorption of surface moisture. Then, they were dried in an oven at 72°C for 48 hours until their weight was constant (DW). The RWC was determined using the following formula: RWC = (FW-DW) / (SW-DW) × 100%. A similar procedure was used to calculate the RWC of the substrate. All previous measures were performed at least five times.

### 2.3 Gas exchange measurement

Gas exchange parameters were measured using a Licor-6400 portable photosynthesis system (Li-Cor, USA). Gas exchange traits of soybean leaves from the top in various treatments were determined between 9:00 and 11:00 am. The net photosynthetic rate (Pn), the stomatal conductance (Gs), the transpiration rate (Tr), and the intracellular CO_2_ concentration (Ci) were recorded simultaneously at room temperature of 25°C, an intracellular CO_2_ concentration of around 400 µmol mol^-1^, an 800 µmol m^-2^ s^-1^ Photosynthetic photon flux density (Shen et al., 2019). The carboxylation efficiency (Pn/Ci) was calculated based on the values of Pn and Ci. The instantaneous water uses efficiency (WUEi) was calculated using the Pn/Tr formula.

### 2.4 Measurement of rapid chlorophyll fluorescence characteristics

Chlorophyll fluorescence is commonly employed to assess the effects of environmental stressors on the photosynthetic efficiency of plants. The fast chlorophyll fluorescence parameters of the middle blades of the 3rd to 5th mature leaves from the apex in various treatments were observed by a multifunctional plant efficiency analyzer (M-PEA, Hansatech, UK) and the variables were estimated according to Cai et al. (2020).

### 2.5 Measurement of antioxidant enzyme activity and reactive oxygen species

Tomato leaf and root samples (0.2 g) were homogenized with a mortar and pestle in 1.6 mL of 50 mM precooled sodium phosphate buffer (PBS, pH 7.8) on ice and centrifuged at 4°C for 20 min. The collected supernatant was used as a crude extract to detect antioxidant enzyme activities. The antioxidant enzyme namely superoxide dismutase (SOD), peroxidase (POD), and catalase (CAT) were determined following the method developed by Kazemi et al. (2010).

Malondialdehyde (MDA) concentration was assessed according to Altaf et al. (2021). Shortly, 0.5 g of leaf and root samples were homogenized in 5 mL of 5% (w/v) trichloroacetic acid (TCA) solution and then centrifuged at 4000 × g at 4° C for 10 min followed by supernatant collection. After that, 2 mL of TCA containing 0.67% thiobarbituric acid (TBA) solution was mixed to the collected supernatant followed by boiled in a water bath (100°C) for 30 minutes and later cooled on ice. The absorbance of aliquot was recorded at 450, 532, and 600 nm using a spectrophotometer. The MDA content unit was expressed as mircomoles per gram of FW.

Hydrogen peroxide content in the roots and leaves of soybean was examined by Wu et al. (2022). In brief, leaves (0.2 g) were sampled and homogenized in 1.6 mL of 0.1% precooled trichloroacetic acid (TCA) on ice and centrifuged at 4°C at 12000 g for 20 min. The supernatant was collected as the crude extract to determine the content of H_2_O_2_.

Superoxide anion production was estimated as indicated by Du et al. (2023) with minor modifications. In short, leaf and root tissues (0.2 g) were ground in 2 mL of 50 mM phosphate buffer (pH 7.8) followed by centrifugation at 12000 × g at 4°C for 20 min. Next, 0.5 mL of 50 mM phosphate buffer (pH 7.8) along with 0.1 mL of 10 mM hydroxylamine hydrochloride were incorporated in 0.5 mL supernatant and later incubation at room temperature for 30 min. Following after incubation, 1 mL of 17 mM sulfanilamide and 1 mL of 7 mM naphthylamine were added into the mixture solution and again incubated for 30 min. The absorbance reading was recorded at 530 nm.

### 2.6 Determination of ABA content

The endogenous ABA content of the soybean leaves was evaluated according to Ma et al. (2022). Briefly, 0.3 g leaf and root samples were homogenized in 3 mL precooled 50% chromatographic methanol (v/v), and the extracted was incubated at 4°C for 12 h, followed by centrifugation at 10000 rpm · min^-1^ for 10 min at 4°C, and the supernatant was stored at 4°C. 2 mL of precooled 80% methanol was added to the residue, extracted at 4°C for 12 h, and centrifuged at 10000 rpm · min^-1^ for 10 min at 4°C. After that, 2 mL of precooled 100% methanol was mixed to the residue and extracted for 12 h, and centrifuged as per the above conditions. Finally, all the extracts were collected and combined, and PVPP (crosslinked polyvinylpyrrolidone) was added into the extract at the rate of 0.2 g·FW^-1^ to adsorb phenols and pigments. After shaking at 4°C for 60 min, centrifuged the mixed solution like the above condition. The supernatant was passed slowly through the C18 column, collected in a centrifugal tube, and then kept in a freeze-drying machine. Thereafter, 2.5 mL of 50% methanol was added to the supernatant for dissolution, and passed through the 0.22 μm organic phase ultrafiltration membrane to determine ABA. The analyses were performed with a Hypersil ODS C18 column (250 mm ×4.0 mm, 5 μm) and a two-solvent system including methanol and ultrapure water (0.5% glacial acetic acid added). The quantitative value of ABA was calculated by an external standard calibration curve method.

### 2.7 Gene expression analysis by RT-PCR

To examine the effect of melatonin on the relative expression of particular genes in soybean leaves under drought stress, total RNA was extracted from soybean leaf tissues using the TotalRNA kit, and cDNA was synthesized after removing genome DNA using TransScript One-Step gDNA Removal and cDNA Synthesis Supermix (Transgen Biotech). After reversed transcription, the cDNA solution was diluted five times and utilized as a template. The specific primers used in this study are enlisted in **Table S1**. The SYBR ^®^ Premix Ex TaqTM kit (Tli RNaseH Plus, TaKaRa, RR420A) was used for qRT-PCR as a master mix, and the reaction system was arranged in accordance with the guidelines. The relative gene expression was quantified following the formula developed by Livak and Schmittgen (2001).

### 2.8 Statistical analysis

All data were statistically analyzed using IBM SPSS 24.0 (Statistical Package for the Social Sciences, SPSS Inc., Chicago, IL, USA). The least significant difference (LSD) test was used to examine the differences between treatments and at least three replications (n=3) were employed for each treatment Differences were considered significant at *P <*0.05. Origin 9.0 software was used to make figures.

## 3 Results

### 3.1 Melatonin improves soybean growth under osmotic stress

Osmotic stress severely inhibited plant growth, as evidenced by leaf desiccation, softening petioles, and plant wilting, while foliar application with melatonin resulted in a significant reduction in plant growth despite water loss and leaf wilting (**Figure S1**). Biomass measurements indicates that osmotic stress significantly reduced soybean plant growth, but melatonin largely counteracted this harsh effect. PEG treatment reduced the fresh weight of both shoots and roots to 77%, and 97%, respectively, than that of the control (**Table 1**). In contrast, while melatonin was incorporated, they were 88% and 126% of the controls and 39% and 40% greater than when PEG treatment alone was used. Similarly, the dry weights of the roots and shoots under PEG treatment were 53% and 44%, respectively, while the application of melatonin increased their dry weight by 66% and 21%, respectively, compared to their corresponding control. Based on dry weight, it was determined that PEG treatment had no discernible effect on the root/shoot ratio but that melatonin treatment resulted in 41% increase over PEG treatment alone (**Table 1**). These findings imply that melatonin has a significant ameliorating effect on the osmotic stress-induced growth inhibition of soybean.

**Table 1:** Interactive effect of melatonin on biomass production of soybean plants under osmotic stress.

Treatment	Fresh weight (g)		Dry weight (g)		Root/shoot ratio
	Shoot	Root	Shoot	Root	
Control	7.8 ± 0.15^b^	2.5 ± 0.08^a^	2.5 ± 0.06^a^	0.45 ± 0.05^b^	0.32± 0.01^a^
Melatonin	8.5 ± 0.19^a^	2.7 ± 0.07 ^a^	2.6 ± 0.08^a^	0.62 ± 0.07 ^a^	0.32 ± 0.02^a^
PEG	3.7 ± 0.12^d^	1.0 ± 0.02^c^	0.8 ± 0.02^c^	0.12 ± 0.02^d^	0.27 ± 0.01^b^
Melatonin+ PEG	5.2 ± 0.18^c^	1.5 ± 0.04^b^	1.3 ± 0.04^b^	0.18 ± 0.04^c^	0.28 ± 0.01^b^

Soybean seedlings cultivated on nutrient solution (Control), seedlings cultivated on nutrient solution and foliar sprayed with 100 μM melatonin for 14 days (Melatonin), seedlings treated with 20% PEG 6000 for 14 days (PEG), and seedlings treated with 100 μM melatonin and 20% PEG 6000 for 14 days (Melatonin+ PEG). At least three biological replicates were measured for each treatment. Difference letters indicate significant variations among the treatments at P<0.05.

### 3.2 Melatonin enhances gas exchange characteristics of soybean leaves under osmotic stress

**Figure 1:**
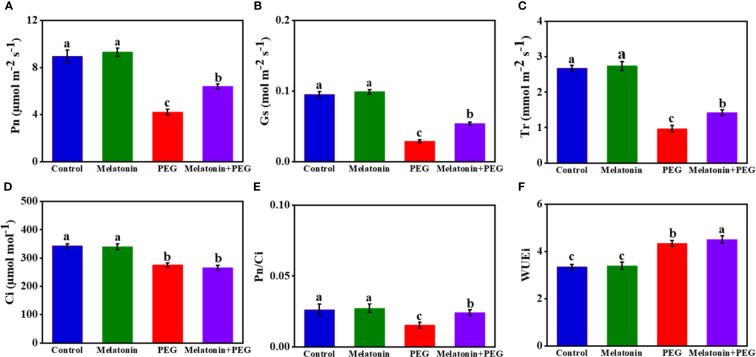
Interactive effect of melatonin on gas exchange parameters **(A)**, net photosynthetic rate (Pn); **(B)**, stomatal conductance (Gs); **(C)**, transpiration rate (Tr); **(D)**, intercellular carbon dioxide concentration (Ci); **(E)**, carboxylation efficiency (Pn/Ci)); **(F)**, water use efficiency (WUE)) of soybean leaf under osmotic stress. Soybean seedlings cultivated on nutrient solution (Control), seedlings cultivated on nutrient solution and foliar sprayed with 100 µM melatonin for 14 days (Melatonin), seedlings treated with 20% PEG 6000 for 14 days (PEG), and seedlings treated with 100 µM melatonin and 20% PEG 6000 for 14 days (Melatonin+ PEG). At least three biological replicates were measured for each treatment. Difference letters indicate significant variations among the treatments at P<0.05.

The gas exchange attributes, namely net photosynthetic rate (Pn), stomatal conductance (Gs), transpiration rate (Tr), respiration rate (Ci), and Pn/Ci were significantly influenced by osmotic stress, and the values of these gas exchange attributes were significantly reduced by 53%, 69%, 64%, 20%, and 42%, respectively, in soybean leaves exposed to PEG stress than in control plants (**Figure 1**). On the contrary, melatonin supplementation reversed these gas exchange characteristics except Ci and increased by 52%, 86%, 46%, and 33%, respectively, indicating that melatonin treatment potentially mitigates photosynthetic capacity under a stressful environment. In addition, melatonin treatment further augmented the WUEi value, which was higher with osmotic stress (**Figure 1**).

### 3.3 Melatonin modulates rapid chlorophyll fluorescence parameters of soybeans under osmotic stress

As displayed in **Table 2**, osmotic stress resulted in a notable reduction in the maximum photochemical efficiency (Fv/Fm) of photosystem II (PSII), which decreased by 19% from that of the untreated plants. However, the application of exogenous melatonin substantially elevated the Fv/Fm value, which increased by 13% in comparison with PEG-treated plants (**Table 2**). The results showed that the PSII parameters of soybean leaves was damaged under osmotic stress, while exogenous melatonin alleviated the inhibition of PSII caused by PEG-stress. In addition, osmotic stress differentially influenced rapid chlorophyll fluorescence parameters, reflecting as increased and/or decreased. The *W*k (the inhibition of oxygen evolving complex at the donor side of PSII reaction center), *M*o (the maximal rate of QA reduction at the receptor side of PSII reaction center), and *φ*_Do_ (the quantum yield of heat dissipation) values were increased in osmotically stressed soybean plants by 57%,174%, and 58%, respectively, compared with their corresponding control seedlings, and the *ψ_o_* (the efficiency that a trapped exciton moves an electron into the electron transport chain beyond QA), *φ*Po (he maximum quantum yield of PSIIreaction center), *φ*RO (the quantum yield for reduction of end electron acceptors at the PSIacceptor side), *φ*Eo (the quantum yield of electron transport), *PI*ABS (photosynthetic capacity indexes which are based on absorption), and *PI*total (photosynthetic capacity indexes based on the total of PSI and PSII reaction centers) values were decreased by 40%, 35%, 63%, 40%, 76% and 69%, respectively in the same treatment conditions (**Table 2**). On the contrary, treatment with melatonin reversed their values under osmotic stress conditions. According to these findings, osmotic stress increases the quantum yield of heat dissipation while decreasing the electron transfer activities of the PSII reaction center, donor side and receptor side, and PSI reaction center. Treatment with melatonin prevents osmotic stress-induced down-regulation of photosynthetic electron transport in soybean leaves.

**Table 2:** Interactive effect of melatonin on chlorophyll fluorescence attributes on soybean seedlings under osmotic stress.

Fluorescence	Control	Melatonin	PEG	Melatonin+ PEG
*FV/FM*	76.8 ± 0.95^a^	77.5 ± 0.88^a^	62.5 ± 0.75^c^	72.2 ± 0.62^b^
*W_k_*	0.46 ± 0.03^c^	0.44 ± 0.04^c^	0.72 ± 0.05^a^	0.53 ± 0.03^b^
*M_o_*	0.68 ± 0.04^c^	0.63 ± 0.02^d^	1.86 ± 0.14^a^	0.95 ± 0.08^b^
*ψ_o_*	0.68 ± 0.02^a^	0.73 ± 0.09^a^	0.41 ± 0.02^c^	0.45 ± 0.03^b^
*ᵩP_o_*	0.86 ± 0.11^a^	0.94 ± 0.13^a^	0.56 ± 0.08^c^	0.76 ± 0.10^b^
*ᵩE_o_*	0.52 ± 0.08^a^	0.53 ± 0.07^a^	0.31 ± 0.11^c^	0.49 ± 0.09^b^
*ᵩD_o_*	0.26 ± 0.05^bc^	0.25 ± 0.04^c^	0.41 ± 0.13^a^	0.28 ± 0.04^b^
*ᵩR_o_*	0.19 ± 0.02^a^	0.20 ± 0.03^a^	0.07 ± 0.01^b^	0.18 ± 0.03^a^
*PI_ABS_*	2.21 ± 0.18^a^	2.28 ± 0.16^a^	0.54 ± 0.08^c^	2.11 ± 0.16^b^
*PI_Total_*	1.45 ± 0.19^a^	1.54 ± 0.14^a^	0.45 ± 0.07^c^	1.26 ± 0.13^b^

Soybean seedlings cultivated on nutrient solution (Control), seedlings cultivated on nutrient solution and foliar sprayed with 100 μM melatonin for 14 days (Melatonin), seedlings treated with 20% PEG 6000 for 14 days (PEG), and seedlings treated with 100 μM melatonin and 20% PEG 6000 for 14 days (Melatonin+ PEG). At least three biological replicates were measured for each treatment. Difference letters indicate significant variations among the treatments at P<0.05.

### 3.4 Melatonin triggers PSII reaction center protein gene expression in soybean leaves under osmotic stress

**Figure 2:**
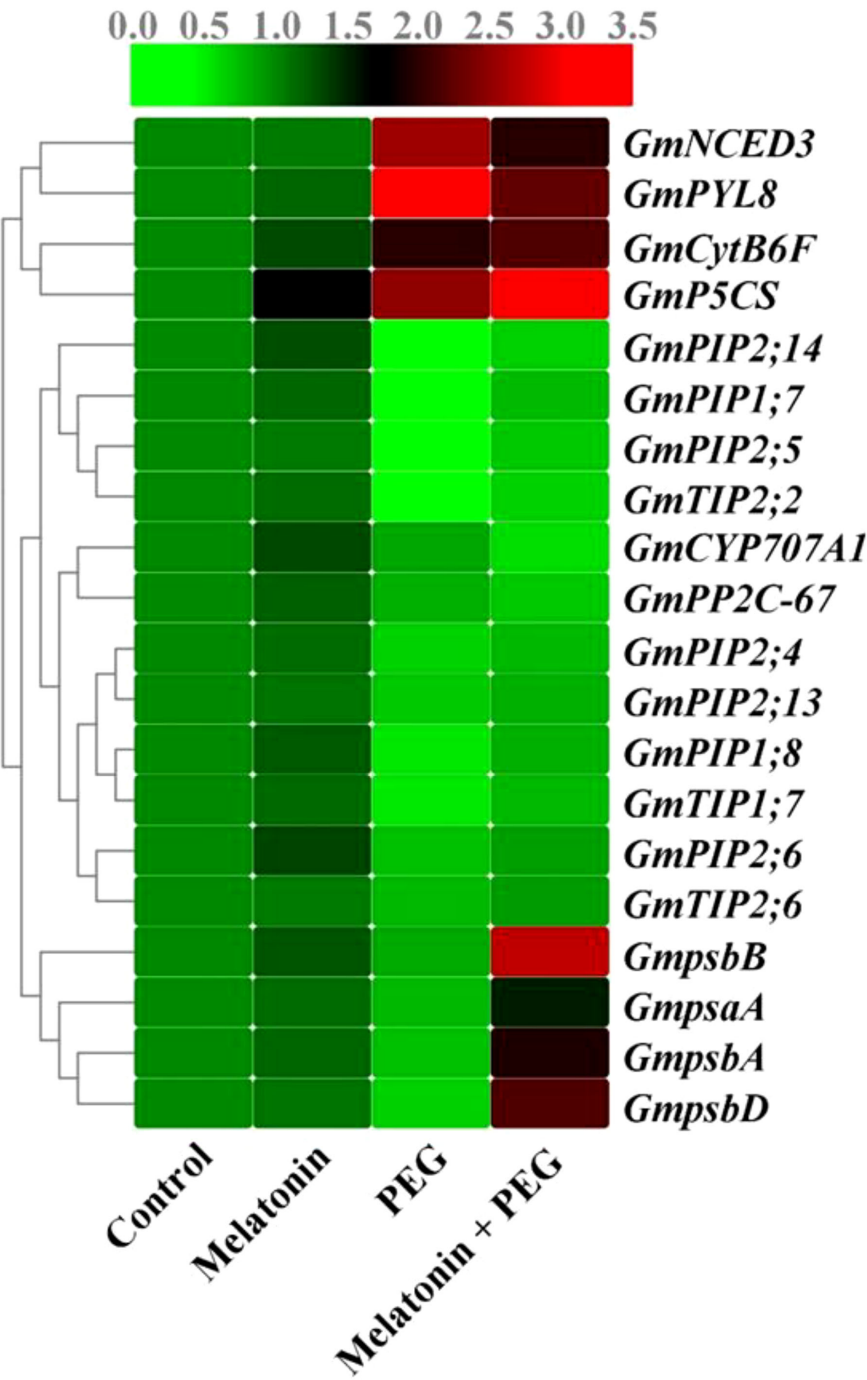
Interactive effect of melatonin on gene expression photosystem, aquaporins gene expression of soybean leaf under osmotic stress. Soybean seedlings cultivated on nutrient solution (Control), seedlings cultivated on nutrient solution and foliar sprayed with 100 µM melatonin for 14 days (Melatonin), seedlings treated with 20% PEG 6000 for 14 days (PEG), and seedlings treated with 100 µM melatonin and 20% PEG 6000 for 14 days (Melatonin+ PEG). At least three biological replicates were measured for each treatment. Difference letters indicate significant variations among the treatments at P<0.05.

The relative transcript abundance of the core protein genes of the PSII reaction center were differentially expressed under osmotic stress conditions. The core genes *psbA* (encoding D1), *psbB* (encoding CP47), *psbD* (encoding D2), and *psaA* expression in soybean leaves under osmotic stress were significantly suppressed, and their expression were reversed after melatonin treatment (**Figure 2**). In addition, the relative gene expression of *CytB6F* was elevated in soybean leaves under PEG stress, and its expression was further up-regulated in melatonin-received plants under the same stress condition, indicating that melatonin has additive effects on core protein gene expression of PSII reaction center under osmotic stress environments (**Figure 2**).

### 3.5 Melatonin maintains relative water content of soybean seedlings and substrate under osmotic stress

After 14 days of osmotic stress, the relative water contents of roots and leaves of soybean seedlings decreased by 37% and 49%, respectively, compared with their corresponding control, whereas melatonin-received plants exposed to osmotic stress were uplifted their RWC content by 31% and 36%, respectively over only osmotic stress plants (**Table 3**), indicating that addition of melatonin significantly increases the water content in soybean under drought stress. In addition, we also determined the substrate water status. The relative water content of the matrix after PEG treatment was higher than that of the control (**Table 3**), while melatonin addition reduces water content in the substrate, indicating that PEG treatment blocks the water absorption so that much more water was left in the substrate, while melatonin treatment significantly improved water absorption of plants so that less water was left in the substrate than PEG alone.

**Table 3:** Interactive effect of melatonin on relative water content in soybean seedlings and substrate under osmotic stress.

Treatment	Root RWC (%)	Leaf RWC (%)	Substrate RWC (%)
Control	72.6 ± 2.54^a^	81.2 ± 2.12^a^	62.5 ± 0.98^b^
Melatonin	73.3 ± 2.98^a^	82.3 ± 2.32^a^	61.5 ± 1.10^b^
PEG	45.6 ± 1.52^c^	41.5 ± 1.81^c^	71.5 ± 1.28^a^
Melatonin+ PEG	59.6 ± 1.85^b^	56.3 ± 1.55^b^	61.6 ± 0.84^b^

Soybean seedlings cultivated on nutrient solution (Control), seedlings cultivated on nutrient solution and foliar sprayed with 100 μM melatonin for 14 days (Melatonin), seedlings treated with 20% PEG 6000 for 14 days (PEG), and seedlings treated with 100 μM melatonin and 20% PEG 6000 for 14 days (Melatonin+ PEG). At least three biological replicates were measured for each treatment. Difference letters indicate significant variations among the treatments at P<0.05.

### 3.6 Melatonin triggers free proline content and its gene expression of soybean seedlings under osmotic stress

**Figure 3:**
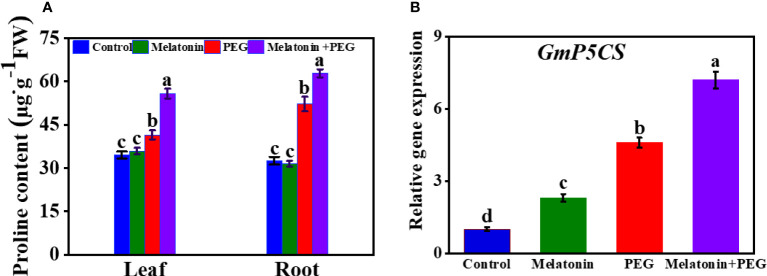
Interactive effect of melatonin on **(A)** proline content of soybean leaf and root and **(B)** *GmP5CS* expression of soybean leaf under osmotic stress. Soybean seedlings cultivated on nutrient solution (Control), seedlings cultivated on nutrient solution and foliar sprayed with 100 µM melatonin for 14 days (Melatonin), seedlings treated with 20% PEG 6000 for 14 days (PEG), and seedlings treated with 100 µM melatonin and 20% PEG 6000 for 14 days (Melatonin+ PEG). At least three biological replicates were measured for each treatment. Difference letters indicate significant variations among the treatments at P<0.05.

Proline is a compatible osmotic solute in plants. Here, soybean plants exposed to osmotic stress increased proline contents in leaves and roots by 20% and 61%, respectively than control plants (**Figure 3A**). The exogenous addition of melatonin further elevated the proline content in both roots and leaves by 1.2- and 1.34 folds, respectively. Consistent with proline content, the core proline biosynthesis gene delta 1-pyrroline-5-carboxylate synthetase (*P5CS*) was highly expressed under drought stress, and its expression was further increased in melatonin-received soybean plants under osmotic stress (**Figure 3B**).

### 3.7 Melatonin balances antioxidant enzyme activity and ROS in soybean seedlings under osmotic stress

**Figure 4:**
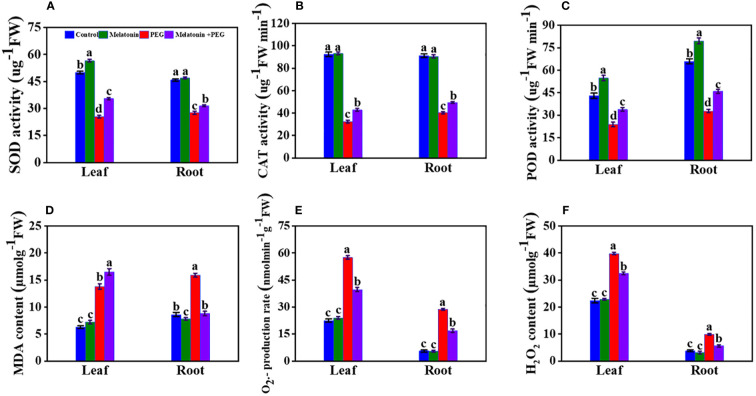
Interactive effect of melatonin on antioxidant enzyme activity of SOD **(A)**, CAT **(B)**, POD activity **(C)**, MDA content **(D)**, O_2_•¯ production rate **(E)** and H_2_O_2_ content **(F)** of soybean leaf and root under osmotic stress. Soybean seedlings cultivated on nutrient solution (Control), seedlings cultivated on nutrient solution and foliar sprayed with 100 µM melatonin for 14 days (Melatonin), seedlings treated with 20% PEG 6000 for 14 days (PEG), and seedlings treated with 100 µM melatonin and 20% PEG 6000 for 14 days (Melatonin+ PEG). At least three biological replicates were measured for each treatment. Difference letters indicate significant variations among the treatments at P<0.05.

After 14 days of PEG treatment in soybean plants, SOD activities in leaves and roots decreased by 49% and 40%, respectively, compared with the control group (**Figure 4A**). On the contrary, the application of melatonin increased them by 39% and 14%, respectively. Similarly, under PEG treatment, CAT activities in the leaves and roots of the plants decreased by 65% and 56%, respectively, compared with control, and its activities increased in plants that received melatonin by 32% and 23%, respectively (**Figure 4B**). Consequently, treatment with PEG significantly decreased the POD activities in the leaves and roots of soybean plants by 45% and 50%, respectively, compared to control plants, and melatonin treatment increased their values by 42% and 40%, respectively (**Figure 4C**). These findings insight that melatonin treatment boosts antioxidant activities under osmotic stress.

PEG treatment significantly elevated the MDA content in the leaf and root of soybean plants by 1.75- and 1.85 times more over control treatment, and the co-addition of melatonin substantially reduced the leaf and root MDA content by 1.15- and 1.02 times more than control plants (**Figure 4D**). Under PEG stress, the rate of O_2_^.-^ generation in soybean roots and leaves was 5.14- and 2.6 folds, respectively higher compared with control plants. Conversely, the production rates of O_2_^.-^ in roots and leaves were lower in melatonin-applied plants when compared to only osmotic-treated plants (**Figure 4E**). These results denote that melatonin co-addition partially but significantly mitigates PEG-induced osmotic stress by lowering the production of MDA and O_2_^.-^ in the leaf and root of soybean (**Figure 4D**-E). However, melatonin treatment significantly inhibits the H_2_O_2_ content in leaves and roots of soybean plants under osmotic stress in contrast to only PEG-treated plants (**Figure 4F**).

### 3.8 Melatonin functions on endogenous ABA and related gene expressions in soybean seedlings under osmotic stress

**Figure 5:**
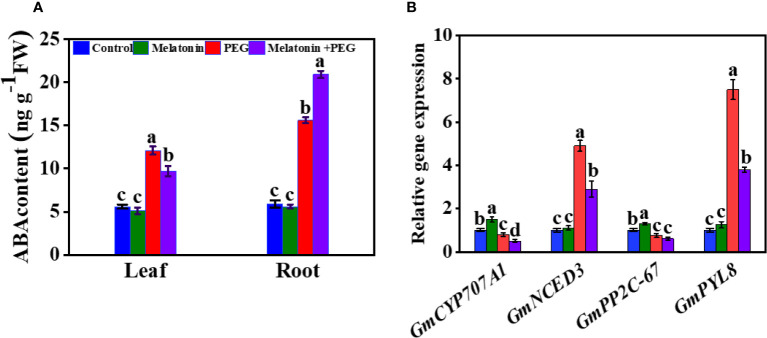
Interactive effect of melatonin on **(A)** ABA content of soybean leaf and root and **(B)** gene expression of ABA metabolism of soybean leaf under osmotic stress. Soybean seedlings cultivated on nutrient solution (Control), seedlings cultivated on nutrient solution and foliar sprayed with 100 µM melatonin for 14 days (Melatonin), seedlings treated with 20% PEG 6000 for 14 days (PEG), and seedlings treated with 100 µM melatonin and 20% PEG 6000 for 14 days (Melatonin+ PEG). At least three biological replicates were measured for each treatment. Difference letters indicate significant variations among the treatments at P<0.05.

ABA is a pivotal component that plays an essential role in the regulation of stress conditions in plants. After two weeks of osmotic stress, the endogenous ABA content was substantially increased in the roots and leaves of soybeans by 164% and 116%, respectively, compared with the control group (**Figure 5A**). Supplementation with melatonin significantly reduced the endogenous ABA content in the leaf but increased the root ABA content of soybean by 34% and 20%, respectively, compared with the only osmotic stressed plant. To understand molecular insight, we also assessed the expression of genes related to ABA metabolism. The relative gene expression of 9-cis-epoxycarotenoid dioxygenase 3 (*NCED3*) and *PYL8* were significantly up-regulated in osmotically stressed plants, and melatonin treatment inhibited their expression under the same stress conditions (**Figure 5B**). In contrast, PEG treatment significantly down-regulated the expression of *CYP707A1* and *PP2C-67* and their expression was further decreased with the supplementation of melatonin under the same stressful environment (**Figure 5B** and **Figure 2**).

### 3.9 Melatonin regulates aquaporin gene expressions in soybean seedlings under osmotic stress

In soybean plants, we sequenced the *PIP*-and *TIP*-related genes in accordance with the conserved sequences of the plant aquaporin genes, including *PIP* and *TIP*, which are confirmed by National Center for Biotechnology Information (NCBI). The relative transcript abundance of *PIP1;7*, *PIP1;8*, *PIP2;4*, *PIP2;5*, *PIP2;6, PIP2;13*, *PIP2;14*, *TIP1;7, TIP2;2*, and *PIP2;6* genes expression were down-regulated by 69%, 55%, 45%, 71%, 35%, 39%, 71%, 56%, 70%, 31%, respectively, compared to their corresponding control treatment (**Figure 2**); however, their expression was up-regulated with the supplementation with melatonin by 119%, 67%, 25%, 110%, 31%, 23%, 93%, 57%, 83%, and 28%, respectively, than the only osmotic stress plants (**Figure 2**). According to these findings, osmotic stress significantly inhibited the aquaporin gene expression in soybean plants, but melatonin treatment greatly reduced this suppression.

## 4 Discussion

Climate change poses significant threats to agricultural regions. Due to shifting climatic circumstances, the negative effects of water scarcity are not just limited to food security but could also result in restrictions in other areas of agricultural production. In this experiment, we investigated the putative role of melatonin on the drought stress tolerance of soybean seedlings. We observed that 20% PEG 6000 led to severe damage symptoms in soybean plants and slowed seedling growth, but the addition of melatonin greatly reduced this retardation effect (**Figure S1** and **Table 1**). These findings support earlier studies indicating that melatonin increases the osmotic tolerance of soybean plants (Ahmad et al., 2019; Altaf et al., 2022). Furthermore, we found that no obvious effect was observed on the root-to-shoot ratio of soybean plants under PEG treatment, whereas melatonin addition significantly increased the root-to-shoot ratio under osmotic stress (**Table 1**). This finding suggests that 20% PEG treatment is obviously enough to inhibit soybean root growth, whereas melatonin significantly lessens drought stress-induced root growth inhibition. The fact that melatonin improved fresh mass substantially more than dry mass (**Table 1**) indicates that melatonin enhances the water status of soybean seedlings exposed to osmotic stress, which can be corroborated by melatonin elevated RWC of soybean seedlings (**Table 3**). Moreover, melatonin also helps to reduce the water content of the growing substrate, indicating that it facilitates plant roots to absorb more water. A major improvement in higher RWC was observed in the melatonin-treated plants and results show the positive effect of melatonin on the water-retaining ability of the plants (Ali et al., 2021; Bhardwaj et al., 2022).

There are two basic explanations of the mechanism by which melatonin enhances drought tolerance in plants, one is increased photosynthesis. As a result, melatonin pretreatment increases leaf chlorophyll concentration and improves the gas exchange and photochemical efficiencies (Arnao and Hernández-Ruiz, 2009; Arnao and Hernández-Ruiz, 2019). Melatonin can also augment the expression of genes related to the Calvin Cycle, including ribulose-1,5-bisphosphate carboxylase/oxygenase small subunit, triose-3-phosphate isomerase, fructose-1,6-bisphosphatase, fructose-1,6-bisphosphate aldolase, and transketolase under heat stress conditions (Jahan et al., 2021). The findings here demonstrated that melatonin lessened the osmotic stress-induced suppression of soybean leaf stomatal conductance and transpiration (**Figure 1**). Among the photosynthetic parameters, stomatal conductance is the rate liming component estimates the rate of gas exchange (i.e., carbon dioxide uptake) and transpiration (i.e., water loss) through the leaf stomata as determined by the degree of stomatal aperture and most vulnerable to osmotic stress. Plant growth is impaired by severe drought stress due to a decrease in stomatal opening, which limits CO_2_ uptake and hence reduces photosynthetic activity (Chaves et al., 2009), and/or from changes in photosynthetic metabolism (Lawlor, 2002). Melatonin on the other hand helps to decrease stomatal closing and increase rate of stomatal opening by means of enhancing stomatal conducting activity (Jahan et al., 2021; Altaf et al., 2022) and thus helps to increase photosynthesis efficiency and biomass production under stress conditions. Melatonin supplementation increases drought tolerance in rapeseed through the enhancement of leaf stomatal conductance and water use efficiency (Dai et al., 2020). The second rationale is to strengthen the antioxidant system. Under drought conditions, tobacco seedlings supplemented with melatonin exhibited higher activities of SOD, POD, as well as other non-enzymatic antioxidants, which scavenged excess ROS and reduced lipid peroxidation production to prevent plant tissues from stress (Zuo et al., 2014; Chen et al., 2018; Iqbal et al., 2018). The findings of the current study supported the previous arguments. The carboxylation efficacy, Pn, WUEi, electron transport activities, and photosynthetic index values of soybean leaves under osmotic stress were all enhanced by applying melatonin (**Figure 1** and **Table 2**). These findings are consistent with melatonin-enhanced leaf photosynthetic performance in other plant species (Wang et al., 2013; Shi et al., 2015; Ye et al., 2016), implying the stimulatory effects of melatonin on the photosynthesis processes under stress are similar in many respects.

The primary protein compound of the PSII reaction center is typically a heterodimer made of D1 and D2 proteins generated by psbA and psbD, respectively (Kiss et al., 2012). The inner light-harvesting complex comprises the CP43 and CP47 proteins, encoded by *psbB* and *psbC*, respectively (Bi et al., 2016). The major photochemical reactions are initiated by these protein complexes, all of which make up the PSII reaction center and control the absorption and transmission of the light energy received by the antenna pigments. The PSII reaction center is the central place of stress damage (Nishiyama and Murata, 2014). In the present investigation, we noticed that the expression of the *psbA*, *psbB*, *psbD*, and *psaA* genes was significantly suppressed by drought stress in soybean leaves, whereas the exogenous application of melatonin entirely reversed this negative effect (**Figure 2**), demonstrating that melatonin overturns the suppressive actions of drought stress on the central protein repair of the PSII reaction center (Jahan et al., 2021).

Our research also corroborated the notion that melatonin increases antioxidant enzyme activity. The result revealed that drought stress enhanced the SOD, POD, and CAT activities in soybean leaves and roots and melatonin supplementation fostered this improvement (**Figure 4A–C**). As a result, melatonin reduced O_2_^-^ generation along with H_2_O_2_ and MDA contents (**Figure 4D–F**) and uplifted the activity of SOD, POD, and CAT. Accumulated evidence showed that melatonin increased antioxidant enzyme activity in different plant species under various stress conditions, including drought stress (Kaya and Doganlar, 2019; Dai et al., 2020; Hossain et al., 2020; Sharma et al., 2020), suggesting that melatonin enhances the antioxidant defenses of soybean leaves to neutralize ROS and inhibits membrane lipid peroxidation production in soybean plants, thereby enhancing plant stress tolerance (**Figure 4D–F**). The physiological implications of melatonin-induced reduction in H_2_O_2_ in soybean leaves under osmotic stress are also unclear. It is widely understood that an increase in H_2_O_2_ in guard cells causes stomatal closure, but melatonin can inhibit ABA-induced stomatal closure in apple leaves (Li et al., 2015) and Arabidopsis leaves (Zuo et al., 2014).

The outcome of water stress often increased endogenous ABA production as a result of both promoting synthesis and inhibiting catabolism (Li et al., 2015; Li et al., 2016). We also observed the accumulation of ABA in soybean plants under osmotic stress (**Figure 5A**). The increase in endogenous ABA content in leaf and root are likely the consequence of stimulation of synthesis and inhibition of catabolism because PEG treatment increased *NCED3* expression and decreased the expression of *CYP707A1* (**Figure 5B** and **Figure 2**) (Li et al., 2015). *PYL8* is an ABA receptor protein gene involved in ABA signaling (Antoni et al., 2013; Kundu and Gantait, 2017). *PYL8* expression was dramatically up-regulated in soybean leaves by osmotic stress (**Figure 5B** and **Figure 2**), showing that transduction of ABA signaling was facilitated during stress. In contrast, *PYL8* expression was down-regulated in leaves when treated with melatonin. This may prove that decrease ABA content in leaf is related with the down-regulation with *PYL8* expression. Kwak et al. (2003) demonstrated that ABA caused an increase in H_2_O_2_ by increasing the expression of the NADPH oxidase gene *Rboh* (respiratory burst oxidase homologue). Hence, the inhibition of ABA accumulation, which in turn increases stomatal conductance, maybe the responsible for melatonin-induced reduction of H_2_O_2_ in soybean leaves during osmotic stress.

Osmotic stress substantially suppressed the transcription of plasma membrane *PIPs* and tonoplast *TIP* in soybean leaves (**Figure 2**), demonstrating that osmotic stress can readily impede water transduction in soybean. Conversely, supplementation with melatonin substantially reduced the suppressive activities of osmotic stress on the transcription of *PIPs* and *TIPs*, revealing that melatonin enhances water absorption and transportation in soybeans, explaining the mechanism underlying melatonin-improved RWC content in plant tissues. These findings help to partially explain why melatonin not only stimulates stomatal opening but also enhances the water balance of plants in drought-stressed environments. The present findings were also confirmed by previous investigations, which concluded that melatonin application triggered the water absorption capacity of the root through the mRNA regulation of *PIP* aquaporins in maize and elevated the *TIP* aquaporins expression in barley under drought stress conditions (Kurowska et al., 2019; Qiao et al., 2020). Plant aquaporin gene expression is stimulated by H_2_O_2_ under stress conditions (Prado and Maurel, 2013; Cai et al., 2020). Moreover, ABA plays a significant regulatory role in promoting rice *PIPs* gene expression (Lian et al., 2006; Ding et al., 2016). In this study, PEG treatment increased ABA and H_2_O_2_ levels in soybean plants, but the plants still exhibited severe damage symptoms and aquaporin gene expression was repressed. As a result, we cannot fully validate the association between ABA and aquaporin gene expression, and it requires further investigation. On the contrary, the positive effect of melatonin is evident. In leaves, melatonin treatment reduced ABA production under osmotic stress, improved stomatal conductance, and enabled CO_2_ entry, which is advantageous for photosynthesis. These effects cause plants to continue absorbing and distributing surface water, ultimately conferring plant drought tolerance.

Proline is an important osmolytes triggers plant adaptability under osmotic stress. Surprisingly, melatonin application can promote higher proline accumulation both root and leaf of soybean seedling even if the intensity of water stress in plants is not as severe as with PEG treatment alone and the core proline biosynthesis gene delta 1-pyrroline-5-carboxylate synthetase expression also increased under osmotic stress (**Figure 3**). Our finding indicates that melatonin-induced proline content in soybean plants is not the product of stress-related damage but rather an active accumulation of osmotic solutes, which may have significant implications for increasing tolerance to drought. These findings, consistent with the earlier study, noted that melatonin-mediated proline activation promotes drought tolerance *via* cell membrane stability and reduced ROS generation (Wang et al., 2019). Collectively, osmotic stress inhibits growth of soybean seedlings, whereas melatonin application mitigates osmotic stress induced growth inhibition by means of increasing photosynthesis efficiency and regulating ABA and aquaporin gene expression.

## 5 Conclusion

In conclusion, our findings show that exogenous melatonin application significantly increased plant biomass specifically root biomass, which might be contribute to improves root water uptake and conductivity, increases photosynthetic activity amid drought conditions, and exerts osmotic stress to soybean plants. Under osmotic stress, the co-addition of melatonin significantly inhibited excess ROS production and reduced MDA levels, thus-facilitates reduction of cellular damage. Osmotic stress greatly decreased antioxidant defense system and melatonin helped to elevated antioxidant enzyme activities. In addition, melatonin prevents ABA synthesis in leaves and increase endogenous ABA content in roots, thereby boosting ABA signaling and generating less H_2_O_2_ accumulation in plant under osmotic stress, and the latter stimulates the up-regulation of aquaporin genes (*PIPs* and *TIPs*) expression, thus increasing the stomatal aperture and transpiration pull. This is advantageous to uptake water, enabling CO_2_ entrance into mesophyll cells and retaining the photosynthetic efficiency of leaves. These studies shed light on the underlying mechanisms of melatonin-mediated improvement of plant drought tolerance. Thus more studies like field trials can be conducted to unravel the efficacy of melatonin in reducing drought-induced growth inhibition under water-limited soil conditions and high-throughput molecular analysis may be the best alternative to get more insightful mechanisms.

## Data availability statement

The original contributions presented in the study are included in the article/Supplementary Materials, further inquiries can be directed to the corresponding author/s.

## Author contributions

XZ: Conceptualization, design of experiment, Methodology, hunting fund; MJ: Conceptualization, performed experiment, Data curation, and Original draft preparation; CJ; Resources, LS: Software, XL: Review and editing. DJ and JN: Revise and editing. All authors contributed to the article and consented to the submitted version.
