# Supplementary material for: Physiological mechanism of melatonin attenuating to osmotic stress tolerance in soybean seedlings
Source: Front Plant Sci. 2023 May 27;14:1193666. doi: 10.3389/fpls.2023.1193666 (PMC10413876; doi:10.3389/fpls.2023.1193666)
Supplement: Supplementary file 1 [file DataSheet_1.docx]

| Gene name | Forward primer | Reverse primer |
| --- | --- | --- |
| *GmPYL8* | CCTCTATCATGTCCCTCCAC | AATATAACCTCCTAGATCCG |
| *GmPP2C-67* | GCTTGTGAAAGCTGCGCTTC | AAACTGGCTGAGCCACCTCC |
| *GmCYP707A1* | TTGAGGTTGCTCCAAAACCC | TGGGATATAGTGTTATGGGC |
| *GmNCED3* | GCATGCCATCGGTGAAACTC | AAGTCGCCGTTGGGTGTTAT |
| *GmCyt B6F* | CGTCCCTCTGTTGTCATGT | GGAGAGGTGATGGTGAAAAGTT |
| *GmpsaA* | AGCAACTCCCTTTTTCACC | GACCCGCTATCAAGAAAAGAAT |
| *GmpsbA* | GCAAACCTATAGCCGCAGA | GGATGGTTTGGTGTTTTGATGA |
| *GmpsbB* | CCCTCTGACCCTGTTCTT | ATATTCCAACCGCCCCAC |
| *GmpsbD* | AACGAAGTCATAGGCACG | CTTTGGGGTTGCTTTTTCC |
| *GmPIP1;7* | CTAAGTGTAGTACCGTTGGGAT | ATGATAGCAGCACCAAGACTAC |
| *GmPIP1;8* | CCAAGACGATGGAAAGGACTAC | TGGCTACAAACTCGGCTATTC |
| *GmPIP2;4* | TCAAGAGCAAGGAGGAGAGTA | AAGGAAGAGAAGGGTTGCTATG |
| *GmPIP2;5* | CAGGTTACAGAGCAAGGTGAA | GCTCTGTACAAGGACCACTTAG |
| *GmPIP2;6* | CTGGGACGACCAATGGATTTA | AGCTTTGATTGCAGAACCTCTA |
| *GmPIP2;13* | TTTGGCAGGAAGGTGGTATG | GTGAGCTCCTCAGCATCAAT |
| *GmPIP2;14* | GATGCTGAGGAGCTCACAAA | GTCTGGTGCTTGTACCCTATAAC |
| *GmTIP1;7* | TCGTGATGACCTTCGGTTTG | AATGGCGATGGGAGCAATAA |
| *GmTIP2;2* | ATCGCTTACAACGAGCTTACA | GACGGAGACACCTACAAACAG |
| *GmTIP2;6* | CACTGGCTATGACACTCCTATTC | ACACCGTGTACACTAATCCAAA |
| *GmP5CS* | CGAACTGAGCTTGCAGAGGGGC | TCGCTTAGCCTCCTTGCCTCC |
| *Actin* | GTATGTTCCTCGAG CTGT | GGACCAGAACGCAAGCT AT |

Table S1 List of oligonucleotides used in this study


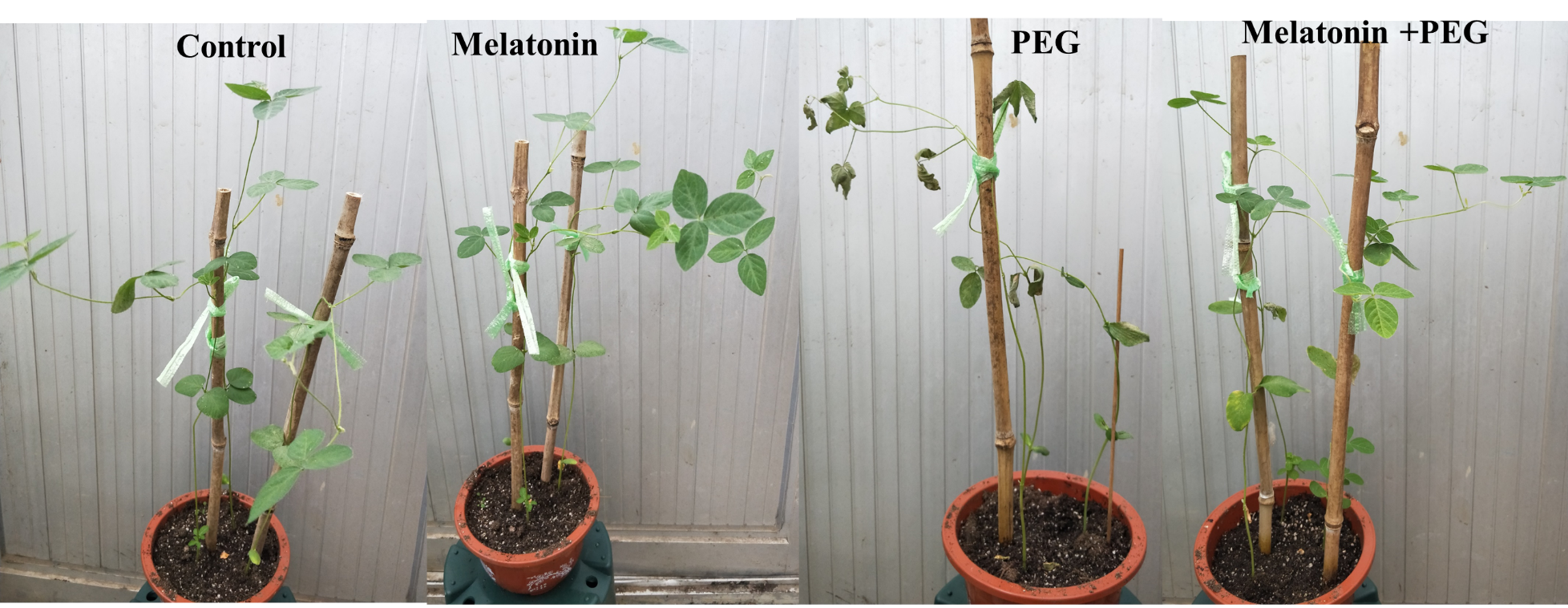


Figure S1 Phenotypic appaearnce of soybean seedlings after osmotic stress. Soybean seedlings cultivated on nutrient solution (Control), seedlings cultivated on nutrient solution and foliar sprayed with 100 µM melatonin for 14 days (Melatonin), seedlings treated with 20% PEG 6000 for 14 days (PEG), and seedlings treated with 100 µM melatonin and 20% PEG 6000 for 14 days (Melatonin+ PEG).
